# vital_sqi: A Python package for physiological signal quality control

**DOI:** 10.3389/fphys.2022.1020458

**Published:** 2022-11-11

**Authors:** Van-Khoa D. Le, Hai Bich Ho, Stefan Karolcik, Bernard Hernandez, Heloise Greeff, Van Hao Nguyen, Nguyen Quoc Khanh Phan, Thanh Phuong Le, Louise Thwaites, Pantelis Georgiou, David Clifton, the Vietnam ICU Translational Applications Laboratory (VITAL) Investigators

**Affiliations:** ^1^ Oxford University Clinicial Research Unit, Ho Chi Minh City, Vietnam; ^2^ Department of Electrical and Electronic Engineering, Imperial College London, South Kensington Campus, London, United Kingdom; ^3^ Department of Engineering Science, Institute of Biomedical Engineering, University of Oxford, Oxford, United Kingdom; ^4^ Hospital of Tropical Diseases, University of Medicine and Pharmacy, Ho Chi Minh City, Vietnam

**Keywords:** signal quality index, electrocardiogram, photoplethysmogram, vital signs, continuous monitoring, open-source, Python toolbox

## Abstract

Electrocardiogram (ECG) and photoplethysmogram (PPG) are commonly used to determine the vital signs of heart rate, respiratory rate, and oxygen saturation in patient monitoring. In addition to simple observation of those summarized indexes, waveform signals can be analyzed to provide deeper insights into disease pathophysiology and support clinical decisions. Such data, generated from continuous patient monitoring from both conventional bedside and low-cost wearable monitors, are increasingly accessible. However, the recorded waveforms suffer from considerable noise and artifacts and, hence, are not necessarily used prior to certain quality control (QC) measures, especially by those with limited programming experience. Various signal quality indices (SQIs) have been proposed to indicate signal quality. To facilitate and harmonize a wider usage of SQIs in practice, we present a Python package, named vital_sqi, which provides a unified interface to the state-of-the-art SQIs for ECG and PPG signals. The vital_sqi package provides with seven different peak detectors and access to more than 70 SQIs by using different settings. The vital_sqi package is designed with pipelines and graphical user interfaces to enable users of various programming fluency to use the package. Multiple SQI extraction pipelines can take the PPG and ECG waveforms and generate a bespoke SQI table. As these SQI scores represent the signal features, they can be input in any quality classifier. The package provides functions to build simple rule-based decision systems for signal segment quality classification using user-defined SQI thresholds. An experiment with a carefully annotated PPG dataset suggests thresholds for relevant PPG SQIs.

## 1 Introduction

Continuous monitoring in ambulatory (Sana et al., 2020) and limited-resourced settings (Nantume et al., 2021) with medical-grade wearables is becoming increasingly widespread as increasing numbers of low-cost devices are available to provide continuously streamed data for long periods of time. Conventionally, physiological signals are recorded continuously for hours, but only the numerals, i.e., the summarized numbers, are reported and used in most analyses. Although powerful insights into disease processes and prognosis have been gained from such methodology, deeper understanding could also be learnt from the signal waveforms themselves as they contain significantly greater information concerning the underlying cardiovascular physiology. Machine-learning and deep-learning technologies now provide us with the ability to analyze these complex waveform data and, hence, the potential to use the electrocardiogram (ECG) and photoplethysmogram (PPG) waveforms to provide pathophysiological insights (Becker, 2006; Elgendi, 2012; Pereira et al., 2020), predict disease progression (Tadesse et al., 2020; Akbilgic et al., 2021; Raghunath et al., 2020), or detect abnormality (Badawi et al., 2021). Analysis of data from low-cost wearable devices, especially in ambulatory patients, can be limited by poor signal quality and noise. Simple-to-use tools to evaluate quality and select appropriate data for analysis are required in order to optimize the potential for devices in improving patient outcomes.

The ECG represents the heart’s electrical activity, transmitted through the body and recorded by electrodes placed on the skin of the torso and limbs. The resulting pattern consists of a baseline and waves, i.e., positive and negative deflections from the baseline depending on depolarization and repolarization activity in the heart. These deflections are named as the P wave, QRS complex (Q wave, R wave, and S wave), and T wave. The PPG records the changes in peripheral blood volume, also in the waveform pattern, by measuring the light intensity (through or reflected) using a sensor placed on the skin of various body parts such as the ear or fingertip. The PPG waveform contains a pulsatile (“AC”) component attributed to change of blood volume with each heartbeat and a baseline component (“DC”) varying at low frequency attributed to autonomic nervous system activity. Both ECG and PPG, recorded by either bedside monitors or low-cost wearable devices, are prone to noise and artifacts. The main causes can be categorized as physiological, (e.g., skin movement or muscle contraction) and non-physiological (e.g., ongoing electrical stimuli, device displacement or signal loss due to Bluetooth disconnection) (Nagai et al., 2017; Lee et al., 2020; Pollreisz, 2022; Seok et al., 2021). In our experience using both finger tip oximeter and patch ECG monitor, loose sensor contact and Bluetooth disconnection, are among the most frequent causes. The timely identification of noisy segments is essential for both monitoring and downstream analyses.

Although many signal quality indices (SQIs) and methodologies have been reported for both PPG and ECG (refer to Table 1), an open-source unified access to a wide range of SQIs does not exist yet. In addition, a powerful toolbox such as the Cardiovascular Signal Toolbox published in PhysioNet is only released in MATLAB. Furthermore, the Toolbox’s scope does not concentrate on deriving the SQI scores but extracting features for analysis.

**TABLE 1:** List of well-known SQIs available in the vital_sqi Python package.

SQI name	Type	Signal	Equation or brief description	Per segment	Per beat	Introduced as SQI in
Perfusion	Stats	PPG	$P_{\mathrm{SQI}}=\frac{\left(y_{\max}-y_{\min}\right)}{\left|\bar{x}\right|}\times 100$	Y	N	
Kurtosis	Stats	PPG	$K_{\mathrm{SQI}}=\frac{1}{N}\sum_{i=1}^{N}{\left[\frac{x_{i}-\bar{\mu_{x}}}{\sigma }\right]}^{4}$	Y	Y	Selvaraj et al. (2011)
Skewness	Stats	PPG	$S_{\mathrm{SQI}}=\frac{1}{N}\sum_{i=1}^{N}{\left[\frac{x_{i}-\bar{\mu_{x}}}{\sigma }\right]}^{3}$	Y	Y	Krishnan et al. (2010)
Entropy	Stats	PPG	$E_{\mathrm{SQI}}=-\sum_{i=1}^{N}x{\left(n\right)}^{2}log_{e}\left(x{\left(n\right)}^{2}\right)$	Y	Y	Selvaraj et al. (2011)
SNR	Stats	PPG and ECG	$N_{\mathrm{SQI}}=\frac{\sigma_{\mathrm{signal}}^{2}}{\sigma_{\mathrm{noise}}^{2}}$	Y	N	Elgendi (2016)
Relative power	Stats	PPG and ECG	$R_{\mathrm{SQI}}=\frac{\sum_{f=1}^{2.25}PSD}{\sum_{f=0}^{8}PSD}$	Y	N	Elgendi (2016)
Mean crossing	Stats	PPG	Number of mean crossings within the signal segment	Y	N	
Zero crossing	Stats	PPG and ECG	Number of zero crossings within the signal segment	Y	N	Elgendi (2016)
MSQ	Morph	PPG	Degree of agreement between two distinct peak detector algorithms	Y	N	Elgendi (2016)
Correlogram	Stats	PPG and ECG	Location and prominence of peaks of the signal segment correlogram	Y	N	Pradhan et al. (2017)
Dynamic time warping	Morph	PPG and ECG	Template matching of a single period with a mathematically described ideal period	N	Y	Li and Clifford (2012)

Thus these challenges have provided the motivation for the development of a SQI package that allows multiple options for practioners, especially in Python. The package we have developed concentrates on the assessment of signal quality. Consequently our work increases the available tools for computing SQI. In addition, the SQI is well-categorized into specific groups. We also design a simple user interface for non-expert practitioners to select their preferred settings. In this study, we focus on the ECG and PPG waveforms derived from wearable devices, where noise and artifact are likely to be highest.

For automated signal quality control, we implemented the 74 state-of-the-art SQIs in a lightweight open-source Python package called vital_sqi. The package is used to help researchers obtain signals suitable for analysis of HRV and training of machine-learning models. The package also provides pipelines to execute end-to-end SQI extraction and classification and graphical user interfaces (GUIs) for users of different programming fluency.

## 2 The vital_sqi package

### 2.1 Installation and requirements

The package is built for Python 3.7 and 3.8, which is released through PyPi, and it is can to be installed through Python Package Manager PIP. The requirements include a number of popular Python packages such as NumPy, Pandas, and SciPy for signal processing; pyEDFlib and WFDB for reading/writing waveform formats such as “EDF” and “MIT (Physio.net).” We also inherit R-peak detection and heart rate variability (HRV) computation from py-ecg-detectors, HRV analysis (Champseix et al., 2018), and HeartPy (van Gent et al., 2019). Further installation instruction and requirements can be found in the source at GitHub and the documentation at Read the Docs.

### 2.2 Structure and modules

**FIGURE 1:**
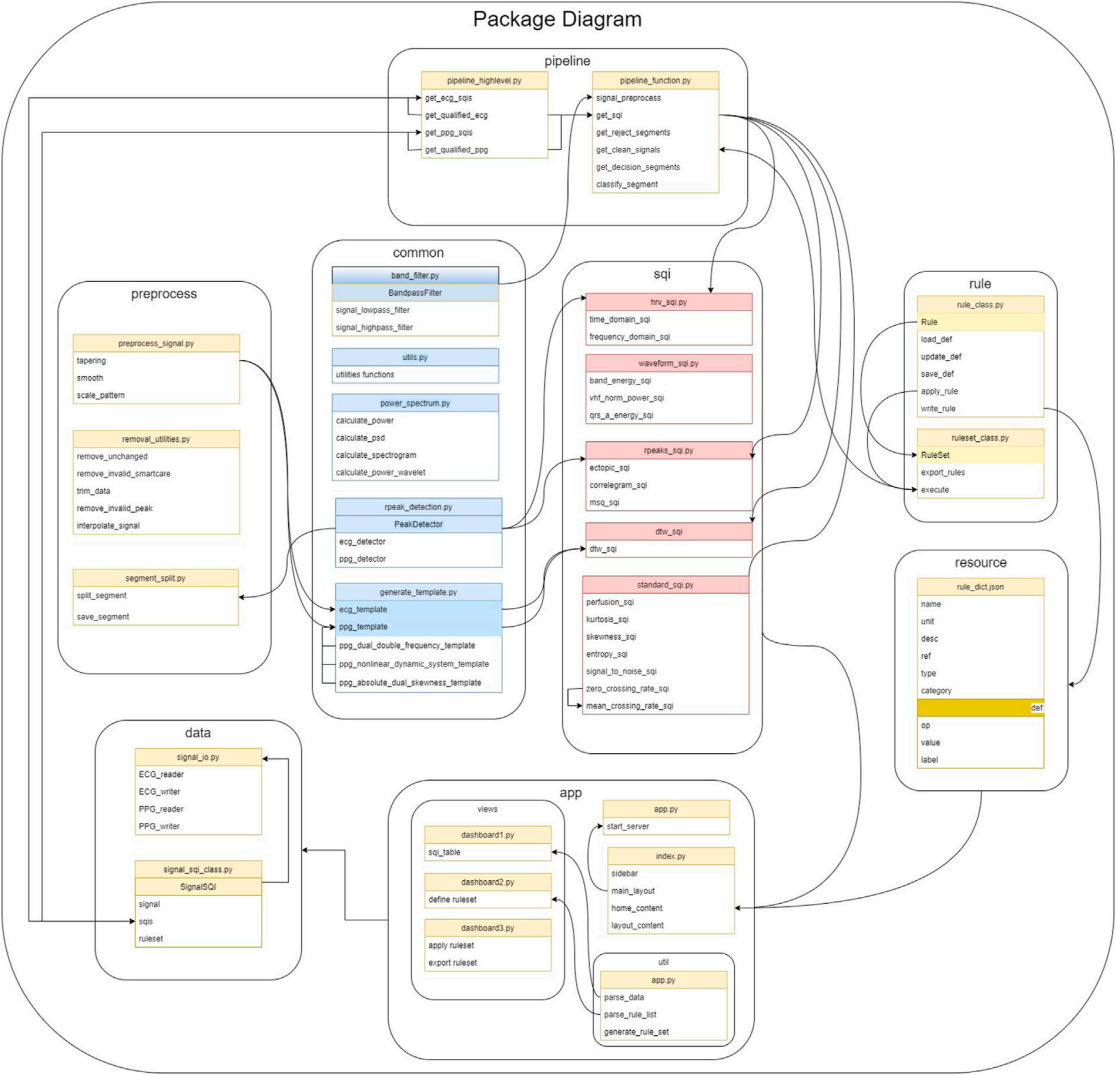
All core SQI functions are implemented in the SQI module. The pipeline module includes functions to load data, preprocess data, and define rules as shown in their respective modules. The pipeline module builds on top of the package to provide general flow from raw waveform to the final result.

The package is structured as a combination of modules for different functionalities as shown in Figure 1. The main workflow contains three steps: data preprocessing, SQI computation, and rule-based decision of signal quality. The modules are designed corresponding to these steps. At any step, the users can introduce results from external computation to use within vital_sqi. To further facilitate flexible use, we provide access to these modules individually and complete pipelines from raw waveform to either 1) SQI table or 2) signal quality classification. All parameters of SQI extraction functions and those ofthe rule-based classification are configurable through SQI and rule dictionaries in the JSON format, allowing clear organization when running quality control experiments.

At the core of the package is the SQI module, in which the 74 selected SQIs are divided into four groups (details in Section 2.3):

• Statistical SQIs such as kurtosis, skewness, and entropy.
• Heart rate variability (HRV)-based SQIs such as SDNN, SDSD, and RMSSD.
• RR interval-based SQIs such as ectopic, correlogram, and MSQ.
• Waveform-based SQIs such as DTW, qrs_energy, and qrs_a.

The usage is built around three classes in *data* and *rule* modules.

• Signal SQI object has attributes for the raw signal, SQI table, and rule-based classification setup. The object is initialized by reading waveforms and is updated at each step.
• Rule object is constructed for each SQI with user-defined thresholds to classify a signal segment as ‘accept’ or ‘reject.’ A number of rule objects are initialized by reading in SQI dictionary from a JSON file.
• Ruleset is a group of rule objects to be executed in order on the extracted SQI table. It is a lightweight, yet efficient, approach to signal quality classification (shown in our experiments in 3.5). The ruleset object is also initialized from a JSON file. Templates of these JSON configuration files, with our recommended thresholds, are provided in the *resource* module.

The two modules of *Preprocess* and *Common* feature share supporting functions to prepare the signal for SQI extraction such as filtering, trimming, or R-peak detection. Last, we provide a web-based GUI, which can easily be used to construct rule and ruleset and execute them on an input SQI table. Coupled with the provided pipelines in the *Pipeline* module for SQI extraction, this *Application* module allows users to quickly obtain quality indices and separate accepted and rejected signal segments.

### 2.3 Signal quality indices

Aiming to separate usable from unusable signal segments, the SQIs implemented in this work have been selected to allow flexible usage, suitable for classification of signals both before and after preprocessing. Therefore, the first step is signal segmentation either by signal duration or by beat, i.e., each segment is equivalent to a cycle (PPG) or QRS complex (ECG). The package features seven peak detectors as shown in Table 2.3.1. The available SQIs are divided into four categories, corresponding to four modules as explained later.

#### 2.3.1 Peak detectors

A total of seven different peak detectors are available for selection when processing data using the vital_sqi package. These are briefly summarized in the table as follows:

**Table:** 

Algorithm ID	Peak detector name	Input parameter	Source
1	Adaptive threshold		Shin et al. (2009)
2	Count origin		Schäfer and Kratky (2008)
3	Clustering		
4	Slope sum		Zong et al. (2003)
5	Moving average		Elgendi et al. (2013)
6	Default SciPy		Virtanen et al. (2020)
7	Billauer algorithm		Billauer (2009)

Users are encouraged to experiment with different peak detectors when working with a particularly noisy datasets. The performance of each detector is influenced by the type and extent of prior preprocessing of the dataset.

#### 2.3.2 Statistical SQIs

Statistical SQIs analyze the signal trends within the segment, providing features of the underlying probability distribution. The implemented SQIs were built on the previous work of Elgendi (2016). The utilization of statistical SQIs to determine PPG signal quality has been significantly researched in the last decade, showing promising performance when distinguishing between the acceptable and unfit signal segments (Selvaraj et al., 2011; Krishnan et al., 2010; Elgendi, 2016). Combining the statistical SQIs with other SQIs provided in this package further improves their cited performance and allows researchers to fine-tune their signal quality selection criteria. For the full list of available statistics-based SQIs, refer to Table 1.

#### 2.3.3 Heart rate variability-based and RR interval-based SQIs

In other modes of operation, the accuracy to identify serial R waves (for ECG) or systolic peaks (PPG) within the signal is examined. RR interval-based SQIs obtain consensus between different methods of detecting RR peaks.

Beyond pure SQI calculation, the HRV indices are calculated from the RR signal, which can allow inference regarding the functionality and control of the heart and nervous system. Using the package, HRV is derived by a sequence of processes: filtering, resampling, peak detection, removing false peaks, and extracting features. These steps are necessary as poor signal quality can lead to inaccurate labeling of R waves and inaccurate HRV indices. Specifically, HRV features in the frequency domain and time domain are distinguishable between normal and ill patient and non-observable in human, which refers to bad quality.

#### 2.3.4 Waveform-based SQIs

In computing these indices, the signal patterns are compared with the standard ECG and PPG patterns. The dynamic time wrapping method uses the distance cost as a similarity score between a single period of the ECG/PPG signal and the generated period Orphanidou et al. (2014). The package also implements other SQIs which evaluate the power energy on the bands of the QRS complex Marco et al. (2012). Other SQIs mentioned in this section evaluate the peak-to-nadir amplitude in the ECG and the systolic-diastolic amplitude ratio in the PPG.

### 2.4 Decision ruleset

As vital signals can be collected from different devices with different modes of operation, a winner-take-it-all method is unfeasible in making signal quality decisions. In practice, the statistical SQIs are appropriate to the longer segments of the signal but highly sensitive to noise and artifacts, which makes selection of the optimal length important. On the other hand, morphology-based SQIs are useful for observing the shorter segments, but the results can be misleading if the underlying waveform is not well-segmented. Our package is advantageous in that it allows flexibility in creating a ruleset by combining various SQIs scores and, thus, can be adapted for multiple scenarios. User-defined rules are set as a chain of SQIs scores using a simple-to-use graphical interface.

## 3 Recommended workflow and experiments

In this section, we present a step-by-step demonstration of the package. This workflow is already designed in the “high-level” module. Users can either get the SQIs list immediately by importing this module or design their own flow using the other modules. The vital-sqi package is composed of the following steps: preprocessing, segmentation, sqi computation, and rule definition. Both ECG and PPG data are used for this demonstration.

### 3.1 Preprocessing

**FIGURE 2:**
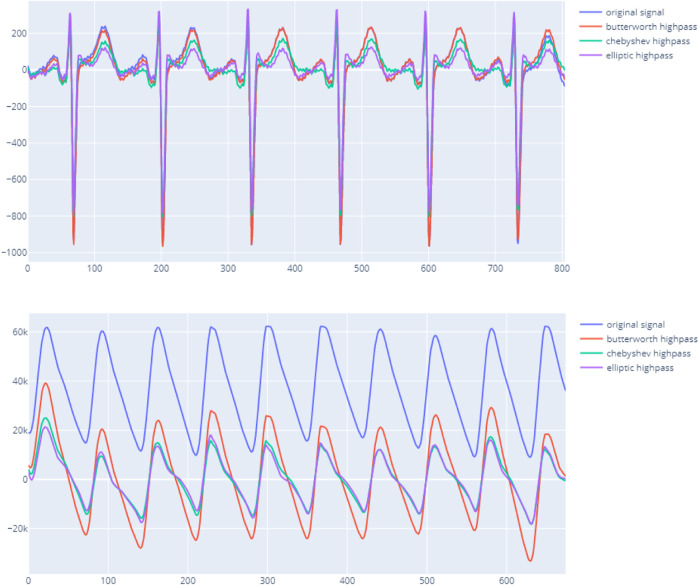
vital_sqi filtering signal using different bandpass techniques on ECG upper and PPG lower.

The main objective of this step is to transform the raw data to match with a standard template of ECG/PPG. First, the signal is checked to remove any missing data or invalid data when reading a given file format. Noise removal is performed by applying the bandpass filter with the appropriate methodology. The Butterworth is the default approach as it provides a good baseline performance and significantly enhances the complex. Figure 2 shows the output using the bandpass method from the package on both ECG and PPG.

In addition to the bandpass filter function, the preprocessing module also implements other techniques to sharpen and expose the signal further such as tapering and resampling. This recommended workflow only filters the signal and removes the missing data. Other preprocessing functions will only be employed later in the SQI computation steps. At the end of the preprocessing process, the package marks chunks of certainly invalid signals. The package considers any available values, unchanged sequences, and zero-unit values as invalid signals. Only chunks of valid signals are used for computation of SQI scores.

### 3.2 Segmentation

Once clean data are obtained, the signal is split into chunks of shorter intervals. Depending on user preference, the segmentation length can be defined in minutes or seconds. Based on our sample data, we recommend a segment length of 30 s as long enough to compute HRV features while maintaining the SQI scores less vulnerable to noise and artefacts compared to long segments. In addition, using shorter signal chunks improves the accuracy of the subsequent peak detection algorithms.

From this point, users can manipulate the signal further with preprocessing methods such as resampling to enhance the single waveforms. Alternatively, peak detection can be performed in advance to get the local minima, which maps to the troughs of the signal and can serve as splitting points. However, it is not recommended at this stage because of the heavy computational load and ineffectiveness when processing long data sequences. Furthermore, peak detection will be employed later for SQI computation.

### 3.3 SQI computation

As explained in Section 2, the SQI can be computed either using the entire segment or single beats. This step produces a matrix of SQI scores of each segment by applying all possible SQI computations. The SQI module implements all of the reviewed methods and categorizes them into hrv_sqi, standard_sqi, and rpeaks_sqi submodules. Since computing all of the available SQIs is time-consuming, users can select a smaller subset of indices. However, we strongly encourage using at least two SQI techniques from each of these subpackages and at least two that use single beats to preserve the accuracy.

**FIGURE 3:**
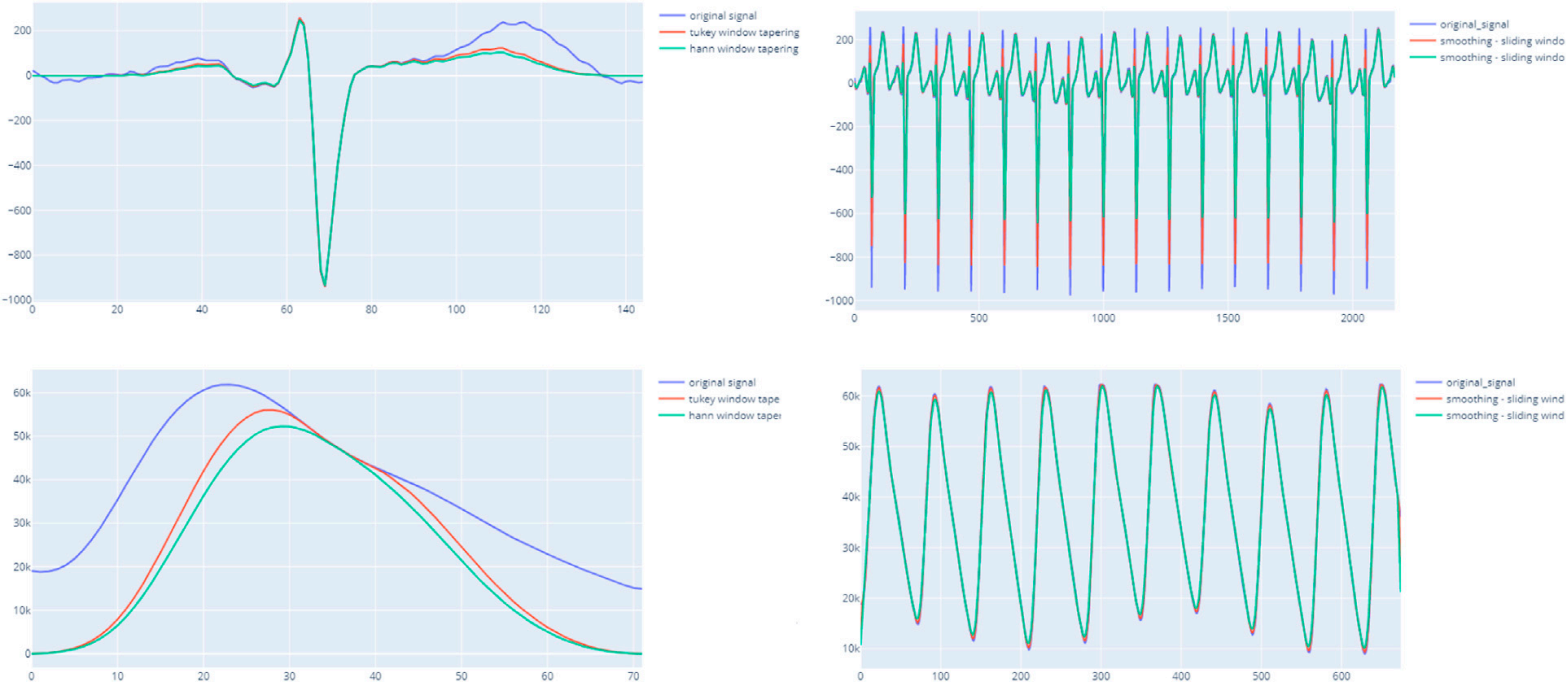
ight-hand side figures illustrate the output of the entire segment when applying the smoothing window with ECG and PPG. The left-hand side indicates a clearer beat morphology of PPG when applying the tapering technique and smoothing windows.

In case of a single-beat algorithm, the mean and standard deviation of all beats within the segment are calculated to contribute to the matrix scores. In detail, the peak detection methods on ECG or PPG are applied in each chunk to extract beats. The extracted beats are transformed by resampling, tapering, and smoothing window techniques to enhance and unify the beat baseline. Figure 3 indicates the importance of enhancing beats before computing SQIs. Particularly, the HRV SQIs are highly sensitive to the appearance of any abnormal spikes. By enhancing the signal, critical points are easier to detect and, hence, increase the accuracy. In addition, the zero crossing rate, mean crossing rate, and dynamic template indices also require unification of the baseline. The computed matrix is then input to the final decision ruleset.

### 3.4 Rule definition

**FIGURE 4:**
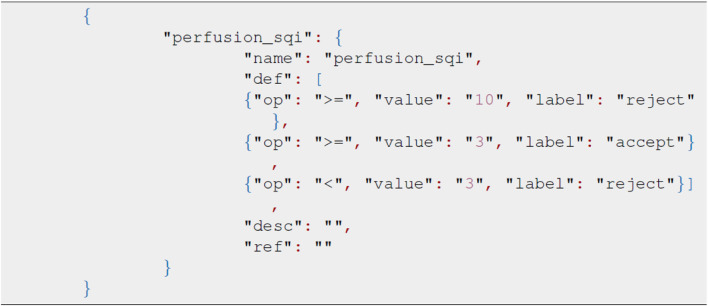
Sample rule definition in the JSON format.

The package defines the ruleset in the JSON format with the key node being the same as the SQI method. Each child node describes the acceptance threshold, score, and relevant decision. The sample JSON format in Figure 4 represents the rule definition using the kurtosis, skewness, and entropy scores as the decision node. The order of selected SQIs can be modified through the JSON file accordingly. In this package, we also provide the recommended threshold for specific SQI scores. The recommended JSON is located in the test_data module, and the values given are derived from our real dataset. When applying numerous SQI scores, modifying JSON can be time-consuming and impractical, and therefore, we have developed a GUI for ruleset modifications.

The GUI is launched as a local web-based application using the DASH framework and Plotly for visualization. The interface is designed using three main dashboards. The home page is used to read the input SQI matrix and the saved ruleset. The uploaded matrix is displayed on the first dashboard, while the loaded ruleset is presented on the second.

Based on user preference, some or all SQI scores can be selected by toggling the rule name button. Each SQI score can have multiple thresholds, different orders and combinations of rules. The final decision will be verified in the third dashboard that will also output the final decision.

### 3.5 Experiment

The experiment aims to demonstrate how the package could be used to label the signal. With an in-house curated dataset, we derived suggestive thresholds for the included SQIs and evaluated the performance of simple rules in signal quality classification.

#### 3.5.1 Dataset

**FIGURE 5:**
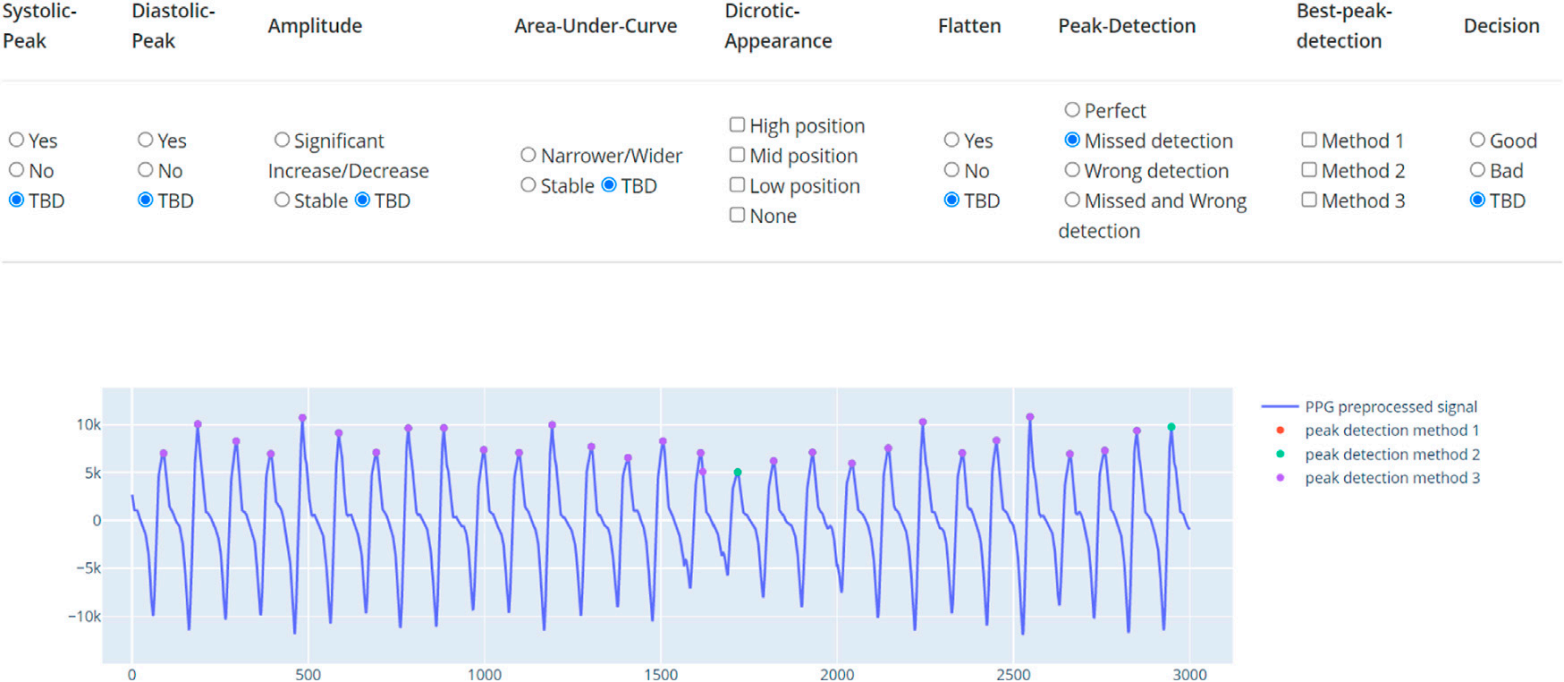
Annotation tool for PPG. The tool indicates multiple factors and the final classification label.

The learning dataset contains the PPG waveforms collected from patients with tetanus treated in the Intensive care unit, lying in a supine position. The device used was the SmartCare oximeter. The study was approved by the ethics committee of the Hospital of Tropical Diseases, Ho Chi Minh City, Vietnam. From 70 long recordings for an an average 20 h, we randomly selected 383 30-s segments. These were double-annotated by doctors as accept or reject based on visual appearance of the waveforms. In the training set, we have a list of criteria to determine the quality of the data segment. The data are annotated by a tool as demonstrated in Figure 5. In this tool, besides the percentage of recognizable peaks, the tool also requires the doctor to mark if the amplitude, width, and trend are abnormal. The annotation process was composed of two rounds. Any uncertain segment in the first round was reserved for the second round. After the first round, 72% of the segments were labeled. In the second round, the opposite decision was made in less than 10% of labeled segments. Most of the changed segments occurred in the initial segments, when the doctor was unfamiliar with the dataset. Although the multi-criterion tool assured high quality annotation, it is time-consuming and also annotates unused factors.

**FIGURE 6:**
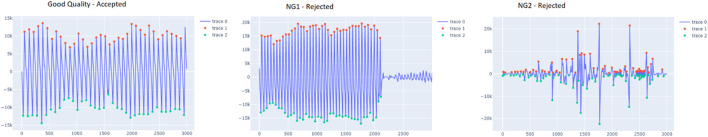
ample segment of the accepted and rejected groups (NG1 and NG2). The red dots indicate the peaks, detected by vitalsqi, while the green dots define the troughs.

Consequently, from the list of well-annotated segments, the waveform-based criteria were simplified to possessing visible systolic and diastolic peaks and the presence of artifacts as presented in Figure 6. The resultant dataset included 273 accepted (A) and 114 rejected segments (NG). The rejected segments were further divided into two groups based on the percentage of cycles with unrecognizable peaks: more than 50% (NG1, 47 segments) and less than 50% (NG2, 67 segments). The same simplified format is applied for the test dataset. This test dataset was composed of 900 accepted segments and 279 rejected segments (210 in NG1 and 69 in NG2).

This training data were then used to determine the thresholds for each SQI. The threshold is defined by a greedy search process, in which the step size is 0.1 quantile unit. The experiments followed the previously described pipeline to obtain the list of relevant SQI scores. Although users can decide the SQIs based on their experience, this package introduces an intuitive yet efficient approach to derive the thresholds. Specifically, the distributions are examined to mark the potential SQI and the appropriate threshold.

#### 3.5.2 Settings

We conducted two experiments, as follows:

1) SQI threshold derivation: For each SQI implemented in the package, we use two methods to determine a threshold that differentiates accept and reject segments. First, the threshold is set as the 95th percentile of the accept histograms. Second, the threshold is determined by a brute force search (step is 0.05 on the quantile) for the most discriminative of the accept and reject segments.
2) Rule building: We searched for the combinations of SQIs that best differentiate the accept and reject segments from the SQIs with the best performances identified earlier; our aim being to show that the combination SQIs (even only two), in a simple rule-based decision approach, result in reasonably good quality assignment.

#### 3.5.3 Results

Using the recommendation in the aforementioned pipeline, we conducted the experiments using a single SQI and a combination of two SQIs. A greedy search was used to verify the performance of all combinations. The final result of the best 10 combinations is illustrated in the table.

**FIGURE 7:**
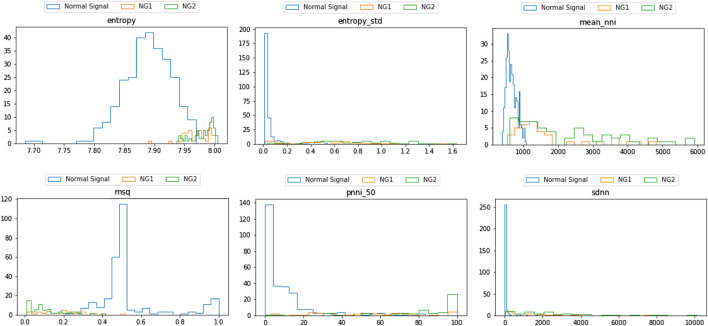
Distribution of different SQI scores with respect to the normal and invalid groups.

As presented in Figure 7, a list of SQIs which compute both per beat and per segment are selected. Looking further into the nominated SQIs, the interpretation is explained as follows:

1) Entropy: This indicates the probability of the appearance of the signal at certain levels. The normal signal of the heart rate is distributed in a normal distribution. In case of an invalid signal, the probability of the appearance of the signal at a certain level is adjusted. Regions that appear rarely can be observed, and it reduces the probability of the normal range. Hence, the information entropy is discriminatory.
2) Mean_nni: This score indicates the mean value of the normal to normal interval (i.e., successive beats). The nni is computed from specific ECG or PPG data. Hence, the result will be extremely vulnerable to any noise and artifacts. In general, the distribution is a normal distribution, and when there is noise, the distribution becomes uniform.
3) MSQ: This SQI computes the consistency of the peak detection algorithm. The signal is shifted n-seconds forward and performs the same peak detection. Assuming the signal is of good quality, the location of the n-second later peak does not vary significantly. However, noise will cause multiple spikes, which potentially result in misreading of the peak and hence inconsistency.
4) Pnn_50: This shows the ratio between NN50 and the total number of heartbeat intervals. NN50 is the number of times successive heartbeat intervals exceed 50 ms, and is believed to be associated with parasympathetic nervous system activity. A bad quality signal leads to increase of the score.
5) SDNN: Among many HRV features, SDNN is the most representative. The score represents the standard deviation of beat-to-beat intervals. Some medical sources use this as the main indicator of HRV, and it is defined that the normal range of HRV is 20–60. As the signal is heavily damaged by the artifact, the SDNN results in greater scores that exceed the normal range.

Following the greedy search process, the results were as follows. According to the distribution, the SQI score differs significantly between the per-beat and per-segment calculation. In terms of the rejection rate, the performance of SQIs per segment is more accurate. Since per-beat analysis mainly depends on beat segmentation, the longer the sequence is, the less accurate the SQI computes. By experimenting with different time lengths, the 30-s segment was observed to be the most appropriate duration. Within this interval, the sequence is long enough to derive meaningful clinical features such as HRV and is also efficient to split by beat.

In summary, using a single SQI as in Table 2, the best 10 combinations using two SQIs compute in per segment, and the rest computes in HRV (which refers to both per beat and per segment). The SQIs are ranked by the AUC rather as the package aims to eliminate as many invalid signals as possible, while retaining a large amount of good-quality signals for later analysis. Generally, all SQIs are effective at identifying the severely distorted signals (R2 group) showing accuracy of above 80%, with most of them (SVSD, SDNN, RMSSD, and SDSD) above 88%. However, the SQIs differ when validating the quality of the R1 group. Most of the SQIs performed with only 60–65% accuracy. Only one SQI (CVSD) reached 70%, while the worst performing SQI (heart rate standard deviation) has only 50% accuracy. Importantly, however, in terms of retaining good-quality signals, all SQIs performed well and retain approximately 90% of valid signals.

**TABLE 2:** Performance of each sqi in classifying a good-quality segment.

Rule	Good accuracy	R1 accuracy	R2 accuracy	Accuracy	Brier	Roc	Fbeta
CVSD	0.9067	0.7095	0.942	0.8024	0.1264	0.8368	0.8761
SDNN	0.9067	0.6667	0.8986	0.7973	0.1366	0.8153	0.8644
RMSSD	0.9078	0.6571	0.8986	0.7981	0.1374	0.81 23	0.8631
SDSD	0.9156	0.6429	0.8696	0.8007	0.1357	0.8072	0.8632
Entropy STD	0.9356	0.619	0.8406	0.8126	0.1264	0.8047	0.8699
Power	0.8889	0.6571	0.8841	0.782	0.1527	0.8011	0.8505
CVNNI	0.9144	0.6333	0.8116	0.793	0.1416	0.7959	0.8566
LF	0.88	0.6571	0.8261	0.7684	0.1629	0.7895	0.8411
HF	0.8489	0.6619	0.9275	0.7566	0.1798	0.7882	0.8314
Heart rate STD	0.9189	0.5286	0.8696	0.8032	0.1535	0.7659	0.8415

In case of combined SQIs described in Table 3, the rejected cases were significantly higher. The 10 most successful combinations detected more than 90% of invalid signals in the R2 group. With the R1 group, the rejected cases were increased, and all of the combinations obtained more than 70% accuracy, with one combination obtaining approximately 80% accuracy. However, this increased performance occurred at the expense of a decrease in accuracy in the good-quality group, where accuracy decreased from 89% to 83%. It is worth noting that the discard rate in the second invalid group was higher than that in the first invalid group, which reflects the fact that signals in the second group are much more distorted.

**TABLE 3:** Performance of the best 10 combinations in classifying the good-quality segment.

Rule	Good accuracy	R1 accuracy	R2 accuracy	Accuracy	Brier	Roc	Fbeta
Entropy STD + CVSD	0.8978	0.7381	0.971	0.799	0.1264	0.8467	0.8785
MSQ + CVSD	0.8489	0.7762	1	0.7651	0.1552	0.8402	0.8604
Entropy + CVSD	0.8367	0.7905	1	0.7557	0.162	0.839	0.8569
SDNN + CVSD	0.8967	0.719	0.9565	0.7964	0.1315	0.8372	0.8729
Kurtosis mean + CVSD	0.9044	0.7095	0.942	0.8007	0.1281	0.8357	0.8747
Correlogram + CVSD	0.8933	0.719	0.9565	0.7939	0.134	0.8356	0.8709
RMSSD + CVSD	0.9	0.7143	0.942	0.7973	0.1306	0.8353	0.873
Zero crossing rate + CVSD	0.9033	0.7095	0.942	0.7998	0.1289	0.8352	0.8741
Kurtosis median + CVSD	0.9033	0.7095	0.942	0.7998	0.1289	0.8352	0.8741
CVSD + CVNNI	0.9033	0.7095	0.942	0.7998	0.1289	0.8352	0.8741

Examining the cases of misclassification, the waveform morphology of PPG signals did not fully match with the standard PPG since the diastolic peaks were indistinct. It is worth noting that these still have utility as the heart rate can still be calculated from these as it is still feasible to identify the start and the end of a cycle. This observation is supported by the fact that the selected waveform focuses on per segment, which is not different in this case and resulted in the score of per segment surpassing the threshold. Finally, the use of a very small cutoff quantile indicates the superiority of the selected SQI and feasibility of this package.

In terms of validating the implementation, our package is limited as the original descriptions validated individual SQIs in diverse datasets. Consequently, each group of SQIs had a specific and different approach to validation. As the statistical SQI group is based on statistics algorithms (eg., skewness and kurtosis), these can be sufficiently validated by computing the values correctly on standard distributions. With HRV-based SQIs, were validated by comparing the values computed from other packages (R-HRV and HRV analyses). For the case of waveform-based and RR interval-based SQIs, the validities were evaluated by comparing a sequence of standard waveforms and the same sequence with additive noise.

## 4 Conclusion

The vital-sqi package has been carefully designed to assist health researchers of different backgrounds in carrying out SQI evaluation. The package provides an end-to-end solution from preprocessing to estimation of SQI scores and definitions of the quality of any ECG and PPG segments. This package concentrates on the estimation of various SQIs; yet, it is also flexible for users with different aims, enabling derivation of HRV features, pre-processing of data or detection of peaks.

Our experiment indicates the feasibility of categorizing the valid signals using the package. Instead of requiring users’ deep domain knowledge, the package provides a simple-to-use pipeline with pre-search thresholds for the classification of invalid signals.

Furthermore, this package also allows users to define SQI rules. The good results described on a real-world dataset indicate the feasibility of the package as applying for other clinical trial setup.

The future work maintains the package up-to-date with modern SQIs and enhances the user interface. Although some SQIs describe the characteristics of the waveform or part of the segment, later versions of the package do not redirect to any vital sign analysis. Instead, the package will cooperate with other well-known libraries such as HeartPy or HRV analysis to retrieve the user needs.

## Data Availability

The datasets presented in this study can be found in online repositories. The names of the repository/repositories and accession number(s) can be found at: https://pypi.org/project/vital-sqi/.
