# Supplementary material for: vital_sqi: A Python package for physiological signal quality control
Source: Front Physiol. 2022 Nov 11;13:1020458. doi: 10.3389/fphys.2022.1020458 (PMC9692103; doi:10.3389/fphys.2022.1020458)
Supplement: Supplementary file 1 [file DataSheet1.PDF]

Supplementary Material

1 SUPPLEMENTARY DATA

The data-set used in this work is also included in the package. Researchers can reproduce the paper work with the given data - located at the test_data module or to use their preferred data.

2 SUPPLEMENTARY TABLES AND FIGURES

As most of the scores do not depend on the devices, the thresholds can be reapply to other data sets. Since some of the SQIs score can be applied in both per-segment or per-beat cases, and also have different settings, the tables only present the standard format.

Table S1 and S2 present the recommended lower-bound and upper-bound threshold for SQI compute in per-segment and per-beat criteria respectively.

Table S4 indicates the threshold from the HRV aspects. The unit of each criteria is as the following:

1) In terms of heart rate, the package uses beats per minute (bpm)

2) In terms of the duration between 2 consecutive beat, the package calculates in millisecond (ms)

SQI	Min Threshold	Max Threshold
snr_sqi	-0.01	0.01
perfusion_sqi	33108.87	8917154.7
mean_cross_sqi	0.02	0.07
zero_cross_sqi	0.03	0.07
msq_sqi	0.27	0.99
dtw_std_sqi	0	0
correlogram_peak1_sqi	23	59.22
correlogram_peak2_sqi	43.39	119.44
correlogram_peak3_sqi	68.39	178.66
correlogram_value1_sqi	-0.54	0.98
correlogram_value2_sqi	-0.16	0.98
correlogram_value3_sqi	-0.51	0.95
skewness_sqi	-0.26	0.87
kurtosis_sqi	-1.25	1.17
entropy_sqi	7.73	7.96

Table S1. Threshold for per-segment SQIs

SQI	Min Threshold	Max Threshold
dtw_mean_sqi	0	0
dtw_std_sqi	0	0
skewness_mean_sqi	-0.46	0.97
skewness_median_sqi	-0.47	0.98
skewness_std_sqi	0.02	0.25
kurtosis_mean_sqi	-1.61	-0.37
kurtosis_median_sqi	-1.63	-0.36
kurtosis_std_sqi	0.01	0.42
entropy_mean_sqi	3.16	4.36
entropy_median_sqi	3.16	4.36
entropy_std_sqi	0.01	0.32

Table S2. Threshold for per-beat SQIs. In the reported threshold, we remarked the no effective threshold of dtw criteria (which is dtw simple mode in the latest version). The up-to-date version of the package has treated this criteria respectively

SQI	Min Threshold	Max Threshold
mean_nni_sqi	403.74	1031.03
sdnn_sqi	7.93	676.03
sdsd_sqi	11.3	831.8
nni_50_sqi	0	20.44
pnni_50_sqi	0	67.61
nni_20_sqi	1.39	28.22
pnni_20_sqi	2.26	86.33
rmssd_sqi	11.31	831.94
median_nni_sqi	403.9	1041.1
range_nni_sqi	40	3778.6
cvsd_sqi	0.02	1.01
cvnni_sqi	0.02	0.78
mean_hr_sqi	58.18	143.81
max_hr_sqi	62.36	201.52
min_hr_sqi	10.9	134
std_hr_sqi	1.12	22.2
lf_sqi	0.33	_
hf_sqi	4.23	_
lf_hf_ratio_sqi	0	15.57
lfnu_sqi	0.46	93.92
hfnu_sqi	4.57	99.33
total_power_sqi	8.07	_

Table S3. Threshold for HRV SQIs

Module	peak_detection	dtw_sqi	hrv_sqi	rpeak_sqi	standard_sqi	waveform_sqi
Libraries	scipy<br>py-ecg-detectors	scipy<br>librosa	numpy<br>hrvanalysis	scipy<br>hrvanalysis	scipy	scipy

Table S4. List of imported libraries in the core vital SQIs modules

Hence, these thresholds do not depend on the devices and can be reapply to other data-set. In the case of low-frequency, high frequency, only 1 threshold is selected as it reflects the 1-side cut-off behavior.
